# THE EMERGENCE AND BENEFITS OF SOCIAL RELATIONSHIPS IN TWO COMMUNITY-BASED DEMENTIA CHOIRS

**DOI:** 10.1093/geroni/igz038.2761

**Published:** 2019-11-08

**Authors:** Andre Smith, Debra J Sheets, Mary Kennedy, Tara Erb, Ruth Kampen, Min Zhou, Chandra Berkan Hozempa, Stuart W MacDonald

**Affiliations:** 1 Department of Sociology, University of Victoria, Victoria, British Columbia, Canada; 2 University of Victoria, Victoria, British Columbia, Canada

## Abstract

Community choir participation for persons with dementia (PwD) confers benefits to health and well-being, including the benefit of socializing which can reduce feelings of loneliness and social isolation. Using the concept of social capital, this study examines the degree to which two intergenerational Voices in Motion choirs facilitate the development of social relationships between PwD, caregivers, and high school students. Data collection involved interviews with 17 dyads of PwD and caregivers, completion of a social relationship questionnaire, and focus groups with a total of 29 high school students. The results show a gradual increase in the level of interactions between all participants, with students in particular interacting more frequently with PwD. Over time, trust and reciprocity emerged within the choirs as more people shared information about themselves. Students’ understanding of dementia changed over time as they learned to appreciate PwD as unique human beings with rich life stories and experiences.

